# Estimation of protein function using template-based alignment of enzyme active sites

**DOI:** 10.1186/1471-2105-15-87

**Published:** 2014-03-27

**Authors:** Brett Hanson, Charles Westin, Mario Rosa, Alexander Grier, Mikhail Osipovitch, Madolyn L MacDonald, Greg Dodge, Paule M Boli, Cyprian W Corwin, Haeja Kessler, Talia McKay, Herbert J Bernstein, Paul A Craig

**Affiliations:** 1 University of California, Irvine, CA, USA; 2 University of Louisville, Louisville, KY, USA; 3 Rochester Institute of Technology, Rochester, NY, USA; 4 Dowling College, Shirley, NY, USA; 5 Rochester Institute of Technology, School of Chemistry & Materials Science, 1 Lomb Memorial Drive, Rochester, NY 14623, USA

**Keywords:** Motif, Enzyme, Catalytic site, Structural homology, Function prediction, Protein data bank

## Abstract

**Background:**

The accumulation of protein structural data occurs more rapidly than it can be characterized by traditional laboratory means. This has motivated widespread efforts to predict enzyme function computationally. The most useful/accurate strategies employed to date are based on the detection of motifs in novel structures that correspond to a specific function. Functional residues are critical components of predictively useful motifs. We have implemented a novel method, to complement current approaches, which detects motifs solely on the basis of distance restraints between catalytic residues.

**Results:**

ProMOL is a plugin for the PyMOL molecular graphics environment that can be used to create active site motifs for enzymes. A library of 181 active site motifs has been created with ProMOL, based on definitions published in the Catalytic Site Atlas (CSA). Searches with ProMOL produce better than 50% useful Enzyme Commission (EC) class suggestions for level 1 searches in EC classes 1, 4 and 5, and produce some useful results for other classes. 261 additional motifs automatically translated from Jonathan Barker’s JESS motif set [Bioinformatics 19:1644–1649, 2003] and a set of NMR motifs is under development. Alignments are evaluated by visual superposition, Levenshtein distance and root-mean-square deviation (RMSD) and are reasonably consistent with related search methods.

**Conclusion:**

The ProMOL plugin for PyMOL provides ready access to template-based local alignments. Recent improvements to ProMOL, including the expanded motif library, RMSD calculations and output selection formatting, have greatly increased the program’s usability and speed, and have improved the way that the results are presented.

## Background

Structural motifs corresponding to enzyme active sites are often highly conserved in functionally related proteins as a result of common ancestry or convergent evolutionary processes [1]. These features can be the basis for inferring function, and computational structural analysis using this approach is now an active area of research [2]. Efforts to this end are motivated by a disparity between the capacity of structural genomics initiatives to generate structures of macromolecules and the ability to characterize these proteins using traditional laboratory methods [3]. The present research is concerned with predicting the function of proteins with known structure and unknown function and with contributing to a better understanding of the structural basis of enzyme activity, primarily by *in silico* methods [4,5]. Numerous algorithms and software applications have been developed for these purposes [6]. There are three major similarity-based approaches to *in silico* function assignment: sequence alignment, backbone alignment and template alignment. Gapped BLAST and Position-Specific Iterated BLAST (PSI-BLAST) [7] and Pattern-Hit Initiated BLAST (PHI-BLAST) [8] are examples of current practice in sequence alignment. Backbone alignment and template alignment are structurally based and allow for the possibility that the same function may be achieved with very different sequences. See [6] for the current state of protein structural alignment including DALI/FSSP [9] and ModBase [10]. More local structural alignment has been done with JESS templates used by the Catalytic Site Atlas (CSA) (http://www.ebi.ac.uk/thornton-srv/databases/CSA/) [1]. The failure of comparison of sequences is not sufficient to preclude similarity of active sites [11], and in some cases, function is determined locally, rather than globally. However, strong similarity of the local three-dimensional structure of active sites may not be sufficient to determine activity *in situ* or even *in vitro,* due, for example, to steric constraints. See, for example, [12]. Thus there is value in use of active-site structural templates as a complementary approach to sequence-based methods and more global methods, but none of the similarity-based methods is sufficient to provide a rigorous determination of activity.

ProMOL is a molecular visualization and analysis tool that uses a template-based approach to alignment. The approach followed in ProMOL is similar to that used in JESS or in FLORA [13] in that it ignores the physico-chemical properties of residues and instead assesses catalytic site structural conservation. It has been available online since 2006 [14] and has been developed as a plugin for the widely used PyMOL molecular graphics environment [15]. ProMOL is being developed collaboratively and distributed freely as open source software (http://sourceforge.net/projects/sbevsl/).

ProMOL uses a library of motif templates derived from active site definitions in the CSA [16] and analyzes spatial relationships and residue identities within a given structure to determine the presence of structural features known to be associated with specific catalytic functions. Features of ProMOL include the abilities

● To make and store motif templates in a growing library of hundreds of existing motif templates,

● To request alignment with subsets of the motifs by template source,

● To view the optimal alignment between a motif template and a query structure, and

● To characterize a structure from the PDB or a structure provided by the user.

ProMOL, in combination with PyMOL, is a standalone application that functions best with an Internet connection (for access to the PDB). Its design as an open source (http://sourceforge.net/projects/sbevsl/files/ProMOL/) plugin to PyMOL is conducive to customization and further development by independent users.

## Implementation

ProMOL was developed for the PyMOL molecular graphics environment in Windows, Mac OS X (including Lion), and Linux. The program is written in Python, and requires Python 2.6, or 2.7. When creating a template for the library, ProMOL uses PyMOL’s API commands to generate a motif template of the active site for an enzyme for which the function is known. When testing a template or using the motif library, ProMOL can search for the motif in a query structure, using the distance selection commands in PyMOL and a set of constraints built-in to ProMOL's Motif Finder module. Additionally, ProMOL contains a module called EZ-Viz that allows the user to interact with the GUI, rather than having to work directly with PyMOL's command line [17].

ProMOL can contain multiple sets of motif templates. The ProMOL release contains a set of motif templates (the P or ProMOL set) from the developers. Once ProMOL has been installed, users can generate their own motif templates (the U or user set). Additional sets are under development.

The P set of motifs was created within ProMOL using catalytic site entries published by the CSA. The PDB ID, EC number, residue name (must be an amino acid), residue number, and chain for each of the catalytic residues were entered in the Motif Maker tab. The process of motif creation is described below. Once the motif was created in ProMOL, it was tested against the template structure to ensure that it worked properly. If there was an exact residue-by-residue match between the motif and the template structure, the motif was saved and tested against known homologs (to search for true positive and false negatives) and random structures (to search for true negatives and false positives). Please note that these numbers provide a limited window on the reliability of structural alignments. In using ProMOL/PyMOL it is also possible to view the quality of the alignment directly, providing a much more reliable method of validation. The motifs were then saved to the P motif set within ProMOL's file structure.

The U set of motifs can be created by any ProMOL user. These motifs are stored in a separate directory on the user’s computer. The location depends on the operating system. See the ProMOL User Guide (http://www.promol.org/home/download/download-now) for specific details.

Initially, matches were ranked based on their Levenshtein distance - a measure of the difference between two sequences [18]. If the two sequences are identical, the Levenshtein distance between them is zero. If there is one difference between the sequences, the Levenshtein distance is one and so on. For example, if a motif contains histidine, serine and glutamate, and the matching region of the query protein contains histidine, cysteine and glutamate, the Levenshtein distance is one.

The Levenshtein distance was incorporated in ProMOL to provide a first level of screening and comparison between the query structures and the motif templates. However, the Levenshtein distance is a coarse measure of structural similarity. To allow more exact quantitative comparison of structural alignments, RMSD calculations were added to ProMOL. RMSD measures the L_2_ norm three-dimensional distance between the atoms in a match; the lower the RMSD between a query and a motif, the better the match. ProMOL can compute RMSD considering alpha carbons only, alpha and beta carbons, or all atoms within a match.

ProMOL works in Windows (including 8), Mac OS X (including Lion) and Linux. The installation differs by operating system; details can be found in the ProMOL User Guide (http://www.promol.org/home/download/download-now). The current release of ProMOL should only be used with PyMOL release 1.3 or higher on all three operating systems.

At present, ProMOL works well with PyMOL in systems that have Python versions 2.5, 2.6 or 2.7 installed. This combination is not functional in systems using Python 3.0 or higher, because PyMOL is normally released against lower versions of Python.

ProMOL can be accessed from the plugin dropdown menu in PyMOL once it is installed properly. The user must download one of the three compressed file formats available: tarball, zip and exe (Windows installer). In each case the contents need to be expanded in the startup folder for PyMOL. The location of the startup folder varies by operating system and PyMOL installation. Examples of the location of the startup folder and the latest details can be found on the web site (http://www.promol.org) and in the ProMOL User Guide.

The motif library for ProMOL is located in the /pmg_tk/startup/ProMOL/Motifs folder. As mentioned earlier, this folder contains the P motif templates (generated by ProMOL). The N set of motif templates (based on NMR structures) and the J set of motif templates (generated by algorithmic conversion of the CSA Jess motifs) is under construction - check the web site for availability. Each motif is a segment of Python code which should not be modified. A motif consists of a series of calls to PyMOL to select residues by type and relative distance. For example, for a serine protease, ProMOL searches for the serine-histidine-aspartate catalytic triad. To start, it sends a call for the specified atoms in the sidechains of all the serines in a protein; as an option, backbone atoms can be added to the atom lists for the desired residues. The next segment of code sends a call for all the histidine atoms that are within a specified distance of a serine residue. The only selections that proceed are those that contain both serine and histidine within a specified distance of each other. At this point, ProMOL sends a call to PyMOL to find aspartate atoms that are within a specified distance of serine atoms and histidine atoms. At the conclusion of this search, ProMOL reports out the atoms that pass the selection process.

**Figure 1:**
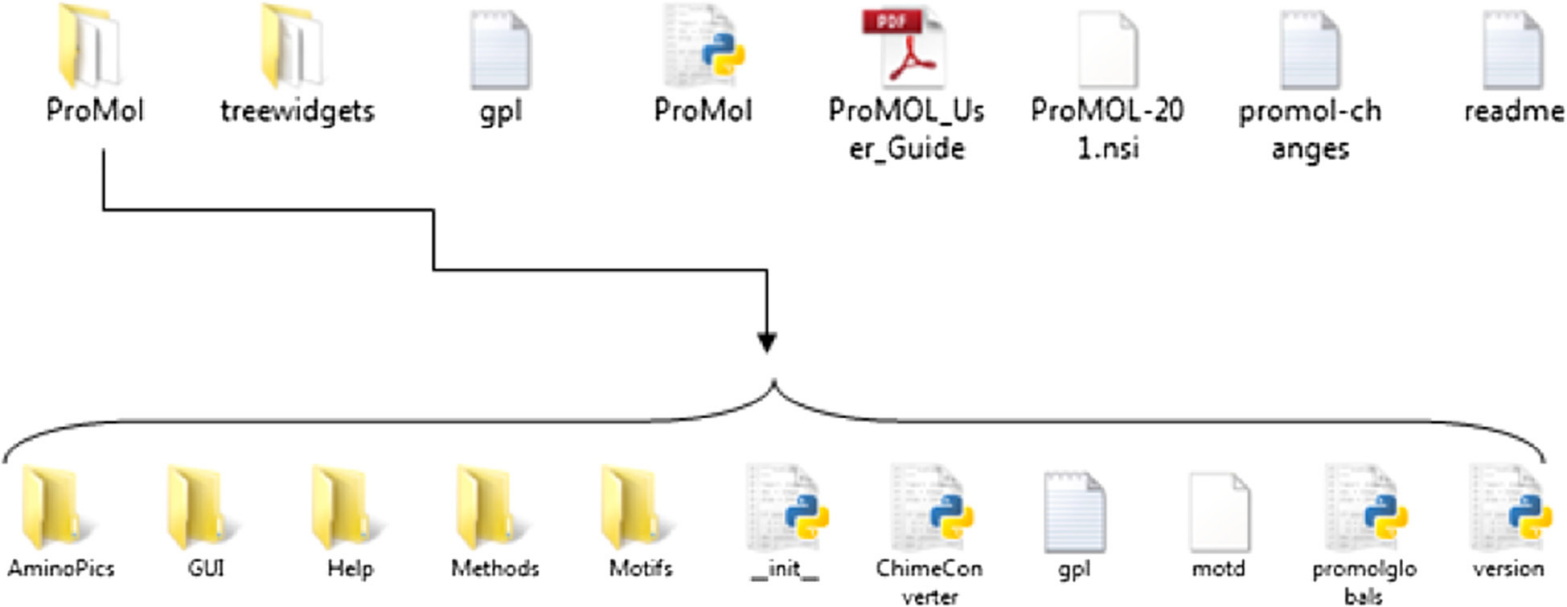
**The directory structure of the release kit for ProMOL.** The items in the first row need to be placed in the pmg_tk/startup folder. The location of this folder is system-dependent, typically under either modules or site-packages as part of the Python installation.

The organization of the ProMOL code is shown in Figure 1. The ProMol.py file controls the execution of the different functions that are located in the ProMOL tabs and buttons. The functions assigned to each tab are described in detail in the ProMOL User Guide. The ProMOL code was created by the authors, with the notable exception of the treewidgets [19], which is required for the RMSD calculation display. Treewidgets is a library for Python and Tkinter for the display of trees containing information based on a wide variety of data structures. ProMOL also accesses the PDB Loader application that ships with PyMOL to load PDB structures over the Internet [20].

**Figure 2:**
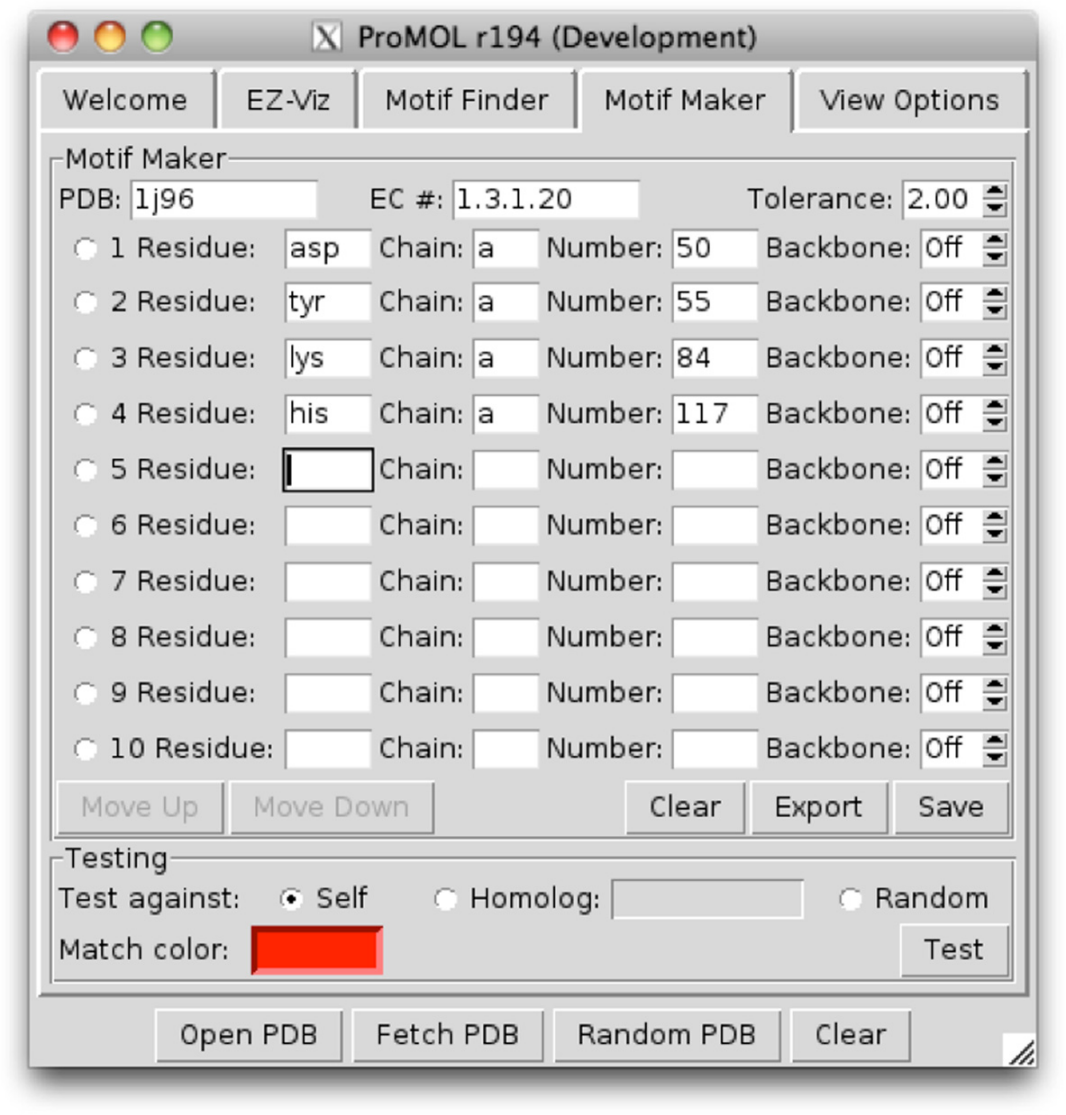
**The ProMOL GUI was created using Tkinter (http://wiki.python.org/moin/TkInter).** Each tab gives the user access to collections of features in ProMOL. The EZ-Viz and View Options tabs allow the user to manipulate the appearance of loaded structures and the PyMOL interface, respectively. Motif Finder is an interface for querying structures against the motif template library. Motif Maker allows users to create their own motif templates for structure analysis.

The GUI for ProMOL (Figure 2) includes four buttons at the base of the window (Open, Fetch PDB, Random PDB, and Clear) and five tabs (Welcome, EZ-Viz, Motif Finder, Motif Maker and View Options). These features are explained in much greater depth in the ProMOL User Guide.

### Motif creation

A library of active site motif templates has been created in ProMOL based on structures that were determined by x-ray diffraction. A motif is a selected set of residues and atoms identifying an active site. In general the relevant active sites have been taken from the Catalytic Site Atlas. The orientation-independent geometry of such a site provides a means of identification of the presence of such a site in another molecule. The motif creation mechanism within ProMOL is based on relative distances among all the active site residues from the template molecules. A full description of motif creation will be provided in a subsequent paper (Osipovitch, in preparation). The templates for the motifs were selected from structures in the Catalytic Site Atlas that were used to create the original JESS motif set [1], plus an expansion to include more complete coverage of the EC classes. The motifs are lists of residue names, atom names and their relative distances that must be matched in an alignment. A tolerance value is included as an addition to the distances to broaden the range of acceptable matches. The default tolerance value for motif creation is 2.00 Angstroms and it can be adjusted as a motif is tested. In addition to the tolerance that becomes part of the actual code of the motif, there is a precision factor that can be used when searching for motifs with the Motif Finder. The precision factor is a multiplier of the distances in the motif, providing an additional way to relax or tighten the constraints for a match. The use of the Motif Finder is described in detail in the ProMOL User Guide, which can be obtained at http://www.promol.org/home/download/download-now.

The motifs were created in Motif Maker and tested as follows:

● The motif template was tested against the template from which that motif was generated using PyMOL/ProMOL to insure that the motif was found in the template structure. If there were any discrepancies between the 3-D image in the Viewer and the motif, several approaches were employed to improve performance: changing the order of the residues in the Motif Maker, adding the backbone atoms to the motif template, and reducing or increasing the tolerance value for the motif template, to eliminate extra residues or to include residues that were omitted, respectively. The motif was then saved.

● The motif template was then tested against the protein’s homologs as listed in the CSA. If there were any discrepancies between the 3-D image in the Viewer and the motif adjustments were made to the tolerance of the motif, which in most cases resolved disparities. After the homolog testing was completed the next set of proteins tested were randomly selected proteins of known function to look for false positives and true negatives.

● Motif templates that were found to be accurate in the motif creation scheme have been added to the Motifs folder in the latest distribution of ProMOL. Ensemble testing of the entire motif library is described below.

### Motif template library

As of 6/21/2013, ProMOL (revision 220) has 181 active site motif templates based on the active sites defined in the CSA. These are labeled with a P prefix in the motifs folder, as they were generated solely within ProMOL. The first ProMOL motif template set was based on the same structures that were used to create the JESS motifs [21]. These structures covered the first two levels of the EC classes found in the PDB, as well as about half of the third classification level (e.g. 3.1.1.x). To increase the accuracy of searches conducted with ProMOL, we searched the PDB for additional structures from which we created motif templates that included all representative structures from the third level of the EC classes.

### RMSD calculations

The first releases of ProMOL used an arbitrary designation of 1 for a perfect match and 2 for an imperfect match (one missing residue or one extra residue). Subsequently, we introduced the Levenshtein distance as the first quantitative measure of active site alignments within ProMOL. This allowed the user to see the number of amino acid differences between the motif template and the match in the query, but it did not contain any information about three dimensional distance differences between the two. ProMOL now can also calculate the RMSD, so that the user can choose to see the three dimensional distances between alpha carbons, alpha and beta carbons, or all of the atoms between the residues in the query and the motif template. This allows for a more quantitative match assessment and easier comparison to other motif-based protein analysis tools.

### Relationship among template-based alignment programs

One measure of ProMOL performance is a comparison against other template-based alignment programs. A number of template-based alignment programs were considered. All of them use conserved three-dimensional motifs to identify similarities in protein structures. Of the programs considered, ProMOL is most similar to 3DMSS-Sites [22,23] and PDBSiteScan [24] because these programs also compare query structures against a library of small motif templates. The results are reported below. ProMOL was not compared with RASMOT-3D [25], which focuses on identifying large conserved folds or with DeepView/Swiss PdbViewer [26], which compares a single motif against the 90% non-redundant set of PDB structures determined by x-ray diffraction.

Since we are using the Catalytic Site Atlas as a major data source for this project, we initially attempted a comparison with their Catalytic Site Search tool, which was down at that time. We instead completed a performance comparison with 3DMSS-Sites version 1.5 (http://bioserv.rpbs.jussieu.fr/cgi-bin/3DMSSSites) [22,23], which uses the motifs from the Catalytic Site Atlas as templates. We also compared ProMOL to PDBSiteScan (http://wwwmgs.bionet.nsc.ru/cgi-bin/mgs/fastprot/pdbsitescan.pl?stage=0), which searches for post-translational modification sites, active sites, and binding sites in 3D structures using a pairwise structural comparison of the 3D structure against sites located in the PDBSite database [24]. The CSA search at (http://www.ebi.ac.uk/thornton-srv/databases/CSS_NEW/), returned to operation during the production of this paper.

Our test set covered all six EC classes with these qualifications for query structures:

● The EC class must be found in the Catalytic Site Atlas and must be defined by a JESS motif [1,16,21].

● The active site must contain at least three residues.

● The EC class must have a definition in the ProMOL library.

## Results and discussion

### Performance summary

ProMOL results are presented as a list of matching motif templates. The list contains the Levenshtein distance for each of the matches and, optionally, RMSD values for the alignment using all atoms, only the alpha carbons, and the alpha and beta carbons. The user can then explore an individual alignment more deeply by checking the “show alignment” box on the Motif Finder, then double clicking on the motif template of interest in the list. This renders the alignment of the query (in red) and the motif template (in white).

The results presented in Table 1 summarize the overall performance of ProMOL with PDB entries that are homologous and non-homologous with the P set of motifs. The average percentage of true positives for all motif templates of the P set is 63%, when a positive is considered true if a match is identified with a query structure that has a matching full EC number (Table 1). The tests were run against all PDB structures with matching EC numbers as of Fall 2012. This is calculated as the proportion of total PDB structures with that EC number which the motif template matched. When run against structures with EC numbers that differ at the first digit, the overall average true negative rate is 81%. If positives are considered true when the query structure matches only the first three levels of EC classification, the overall average true positive rate is 31%. The decrease from 63% to 31% seems a bit puzzling at first, but an example may help here. If the query structures are from EC 1.1.1.1, 1.1.1.2, 1.1.1.3, 1.1.1.4 and 1.1.1.5 and the template is 1.1.1.8, then there is a lower likelihood of finding a true positive than if the query pool is taken entirely from the same 4 digit EC class as the template (1.1.1.8). For true positives where EC numbers match completely, the average RMSD values are 1.675, 1.500 and 1.546 Angstroms, for all atoms, alpha carbons, and alpha and beta carbons, respectively. The standard deviation for all atom RMSD values was calculated as 4.904 Angstroms.

**Table 1:** Summary of motif performance

***Motif set***	***Testing structures***	***Hit rate (%)***	***RMSD All (A)***	***RMSD Alpha (A)***	***RMSD Alpha and Beta (A)***
	Native structure	96.69	~0	~0	~0
P Set	Homologous	62.63	1.675	1.500	1.546
	Non-homologous	18.51	7.19	6.51	6.59

Each motif template of the P set was tested against its homologous structures with the same EC designation, non-homologous structures with a different EC designation, and against its native structure that was used to build the motif template. For each positive hit, a set of three RMSD values was calculated: between all atoms, between alpha carbons, and between alpha and beta carbons. Extremely low RMSD values were recorded for alignments of motif templates with their native structures. The hit percentages for testing against homologous structures represent the true positives rate, and the hit percentages for testing against non-homologous structures represent the false positives rate. These data can also be grouped as true positives (63%), false negatives (37%), true negatives (81%), false positives (19%).

For false positives, based on testing against 200 randomly selected unrelated structures with a different first EC digit per motif, the average RMSD values are 7.19, 6.51, and 6.59 Angstroms with the standard deviation of 10.2 Angstroms for all atoms RMSD values. The lower average RMSD values for positive matches with homologous structures indicate that the average quality of alignments with homologous proteins was higher than that of non-homologous proteins. The average true negative rate of 81% is slightly misleading, because, when a user evaluates a result, they have access to the visual alignment and the corresponding RMSD values. Using this information in concert, users are likely to be able to recognize many false positives as such. The percentages and RMSD values in the preceding two paragraphs are exact (sample size=population size).

**Figure 3:**
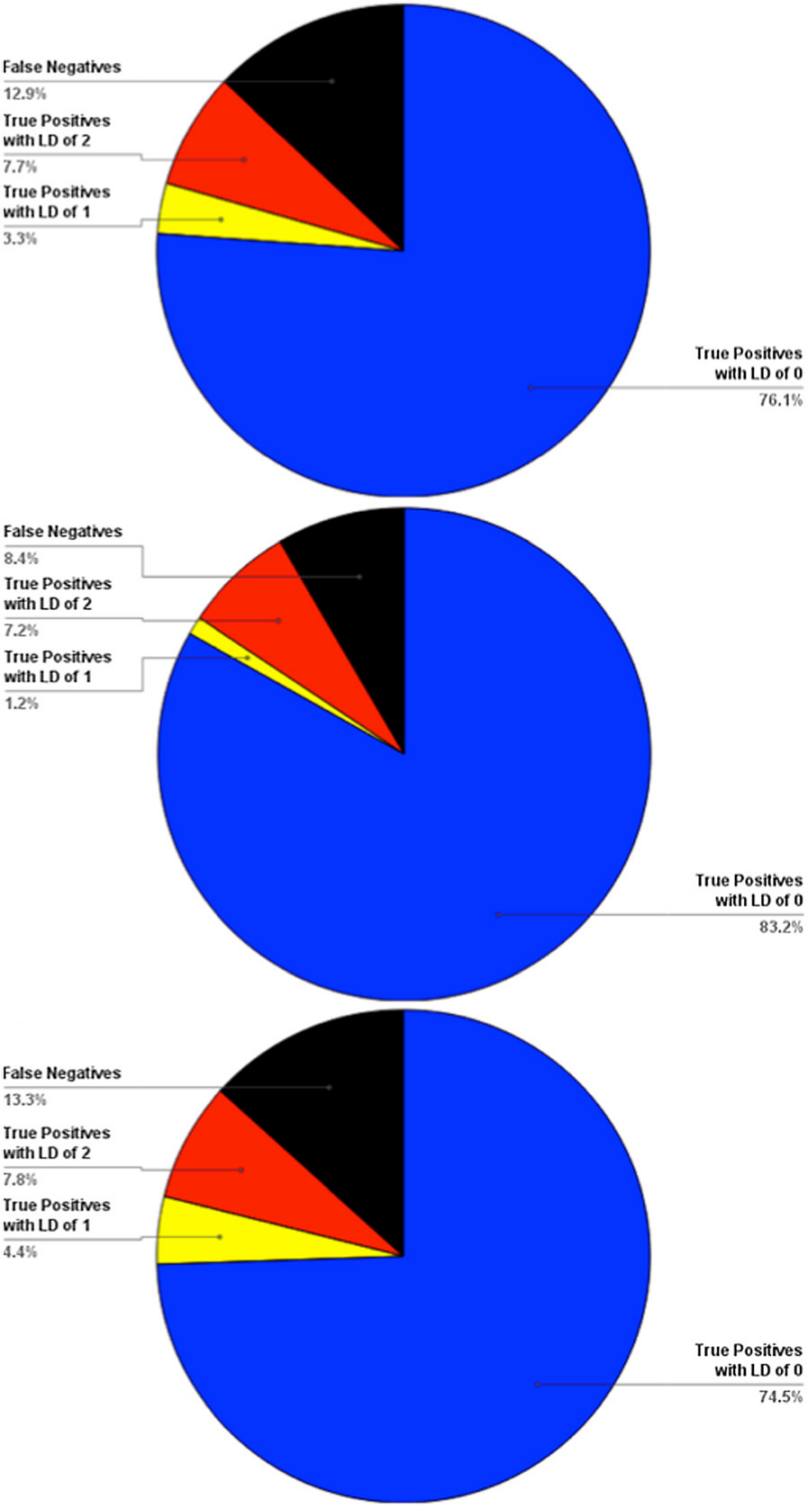
**Levenshtein distance analysis of serine proteases.** Top, Results by Levenshtein Distance for Random Sample of Serine Proteases; Middle, Results by Levenshtein Distance for Serine Proteases that are Motif Template Homologs; and Bottom, Results by Levenshtein Distance for Serine Proteases that are not Motif Template Homologs.

**Figure 4:**
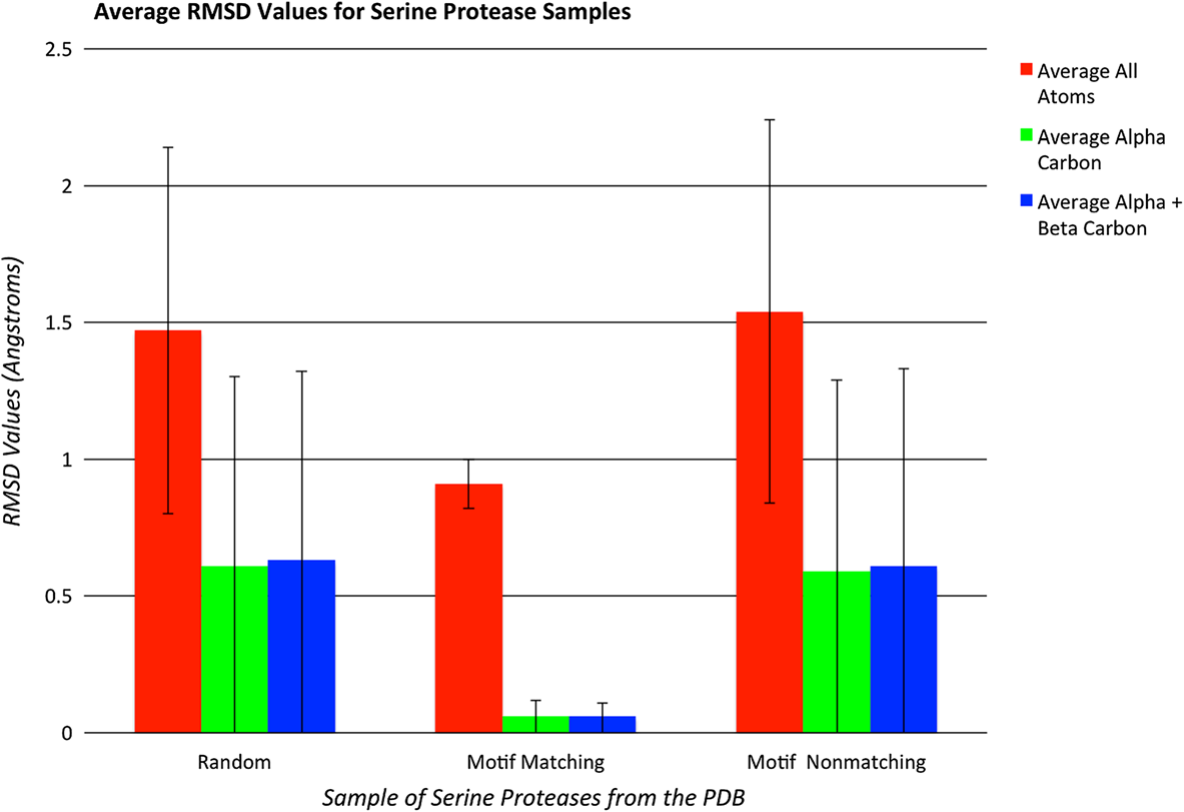
**Average RMSD values for serine proteases.** Left: RMSD values for all serine proteases tested; Middle: RMSD values for serine protease structures with the same EC designation as the structure used to produce a motif template; Right: RMSD values for serine proteases that differ from the motif templates by the last EC level (e.g., motif Template 3.4.21.4 vs. query 3.4.21.5). The error bars reflect one standard deviation from the mean in each case.

An in-depth analysis of the performance of ProMOL was conducted with serine proteases in July, 2012. ProMOL currently includes three serine protease motif templates (1o2u, 3.4.21.4; 1h2x, 3.4.21.26; 1ak9, 3.4.21.62), for which there were a combined 606 structures in the PDB that had one of those three EC numbers. At that time, there were a total of 2098 structures in the PDB which were identified as serine proteases, with EC designations of 3.4.21.*. We found that 87% of the 2098 serine proteases in the PDB match at least one of ProMOL’s three serine protease motif templates. At least one of the templates matches 91.6% of the 606 PDB structures that share their entire EC numbers with one of the motifs. The average Levenshtein distance of all of these matches is 0.2, with a range of 0 to 2, and both subsets (full EC matches and EC matches differing by the last number) also have an average Levenshtein distance of 0.2 and a range of 0 to 2. For all of the matches, the average RMSD values are 1.5, 0.61, and 0.63, for all atoms, the alpha carbons, and the alpha and beta carbons, respectively. For the full EC matches, the average RMSD values are 0.91, 0.060, and 0.062. For the matches differing by only the last number of the EC designation, the average RMSD values are 1.5, 0.59, and 0.61. Average RMSDs were computed as the unweighted means of individual structure-by-structure unweighted RMSDs for the relevant atoms in the motifs that matched atoms in the structure. These average RMSDs are shown in Figure 3. The unweighted estimated standard deviations of those means were used to infer the balanced intervals around the means containing 95% of the population of RMSD values (i.e., the 95% confidence intervals) The inferred confidence intervals are shown in Figure 4. Because RMSD's are inherently cut off to be non-negative, the inferred confidence intervals should only be taken as an approximate visual indicator of the distribution of likely values. The results for serine proteases, when considered relative to the results for all motif templates, indicate that serine proteases are highly conducive to functional assignment on the basis of structural motif analysis. This is likely the result of the catalytic site of serine proteases being highly conserved in both their spatial arrangement and their composition.

In preliminary work for a future study, we examined the sequence alignments for 42 structures that had three or four amino acid alignments with motif templates for which the RMSD was less than 2.5 Angstroms, but for which Clustal Omega [27] showed 25% or less sequence identity. The visual comparison of sites confirmed the good RMSD values on these structures with low sequence homology.

### Performance comparison of template-based alignment programs

**Figure 5:**
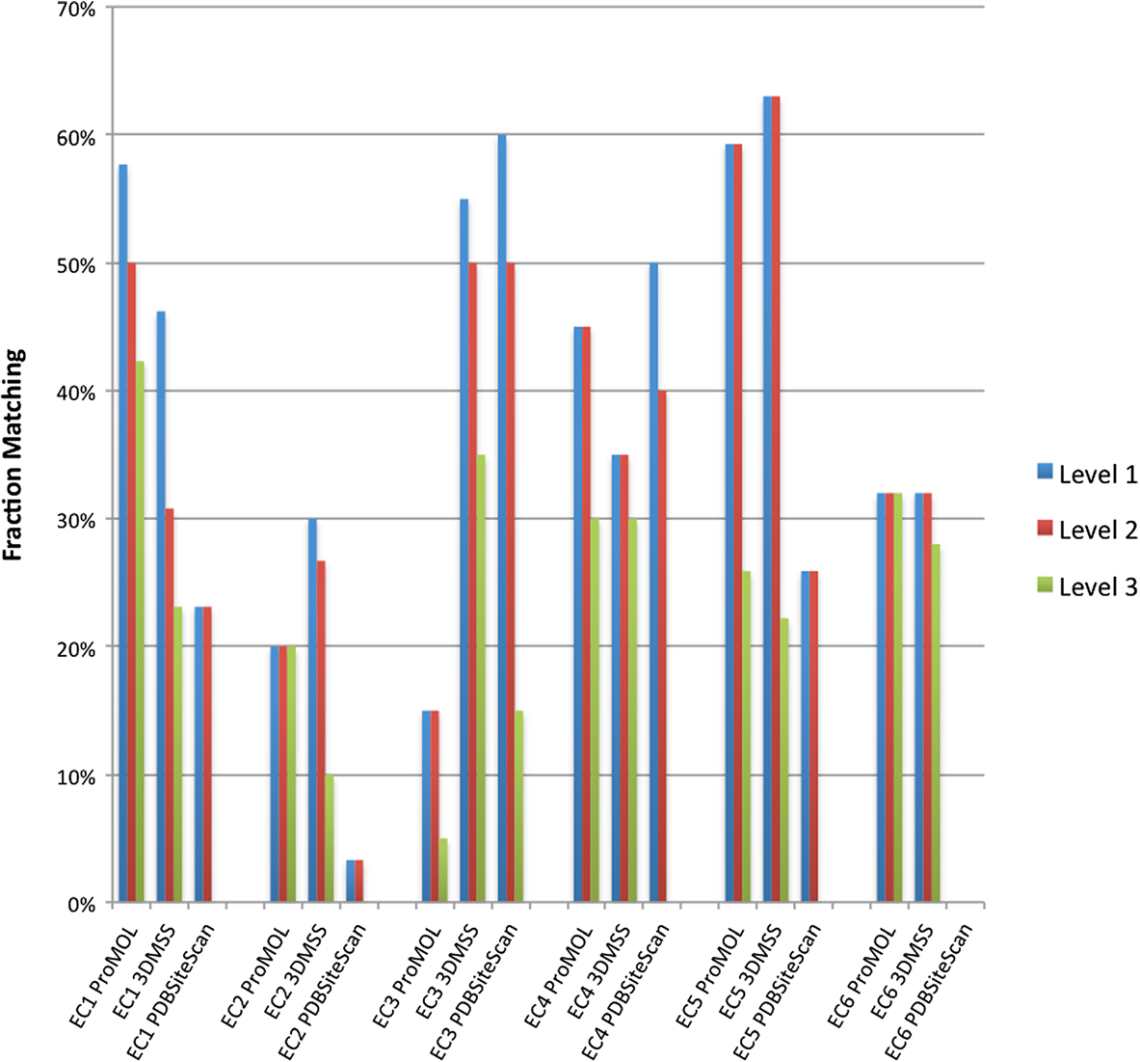
**Comparison of template-based alignments with ProMOL, 3DMSS-Sites, and PDBSiteScan.** Structures from each of the six EC classes were queried using ProMOL, 3DMSS-Sites, and PDBSiteScan. A match is based on the EC Class and residues described in the Catalytic Site Atlas. Level 1 matches shared the first 3 numbers in the EC class; level 2 matches shared all four EC class numbers and level 3 matches include level 2 matches that also share all active site residues. The reported percentages simply reflect the fraction of matches by each method for each EC class. The selection of structures for this test is described under Implementation. The distribution of structures follows: for EC1, a total of 26 query structures from 16 unique EC classes were queried using ProMOL, 3DMSS-Sites and PDBSiteScan. The test sets for the other classes were similar: EC2 (29, 18), EC3 (20, 12), EC4 (23, 13), EC5 (25, 8), EC6 (25, 7).

A total of 148 structures from 75 unique EC classes (distributed over all six EC classes) were queried with ProMOL, 3DMSS-Sites, and PDBSiteScan, and the results are shown in Figure 5. Level 1 matches indicate that the query was correctly identified by the first three numbers in the EC class (e.g., the program reported 1.1.1.85 for a structure with EC number 1.1.1.38); level 2 matches were correctly identified by all four numbers for the correct EC class; and level 3 matches have the correct EC number to four digits, plus they identify all of the catalytic site residues.

The results from the three programs were comparable. 3DMSS-sites provide slightly higher identification by level 1 matching the first three EC numbers at 43%, while ProMOL matched at 39% and PDBSiteScan at 24%. The level 2 matches (matching all 4 EC numbers) for 3DMSS were again marginally higher than those of ProMOL and PDBSiteScan. 3DMSS matched at 39%, ProMOL at 37%, and PDBSiteScan at 22%. ProMOL did a slightly better job with level 3 matches (matching all four EC numbers and containing all active site residues found in the Catalytic Site Atlas). ProMOL matched at 26%, 3DMSS at 24%, and PDBSiteScan barely matched any at 2%. It was clear from the comparison that 3DMSS-Sites is more effective with EC3 and EC5 classes. PDBSiteScan is also successful with EC3 and EC4 classes, but only with level 1 and level 2 matches. ProMOL gave better identifications for EC1 and EC4. The low percentage of identity matches for some EC classes (e.g., EC2, transferases) was surprising in light of the selection criteria for the query structures. To summarize the comparison, 3DMSS-Sites is better at finding the larger families, while ProMOL is better at identifying the exact residues for the active site. In our tests, the results from ProMOL and 3DMSS-Sites were most closely related and differed significantly from the results obtained from PDBSiteScan.

The strengths and limitations of template-based alignments with ProMOL can be summarized as follows:

### Strengths

● A good place to start when assigning the function of a protein structure

● Coverage of all 6 EC classes

● ProMOL/PyMOL operates well on standard laptops/desktops running Windows, Mac or Linux.

● It is possible to run multiple structures in batch mode on ProMOL and collect the results at a later time.

● The interface offers flexibility for data collection (RMSD is optional) and motif template selection.

● The motif maker allows users to construct their own motif template libraries. This can be enzyme active sites, but in fact could include any motif consisting of collections of closely spaced amino acids in a protein.

● Visual confirmation of alignments

● Useful information: Levenshtein distance, RMSD

● Works well with certain classes (EC1, EC4, EC5)

### Limitations

● The rates of false positive and false negatives are too high for strictly automated analysis.

● Structural homology of active sites is not fully determinative of activity. It still needs to be complemented with other bioinformatics tools, such as BLAST, to predict substrate specificity.

● The program runs fairly slowly (database implementation will relieve this).

● The true positive rate is particularly low with EC2 or EC3.

● The templates are all based on enzyme active sites, so it does not account for sites involved in ligand binding, sites for protein interactions with other proteins, nucleic acids, carbohydrates or organelles.

● Our motif templates all include side chain data. Due to the limited data resolution of most macromolecular structures, these data are much less reliable than Calpha, Cbeta information in structures in the PDB [1].

### Future plans

Database- An SQL database of search results is being developed to mitigate the computationally intensive nature of searching many query structures against many motif templates, as well as to limit the number of searches that need to be performed. This new implementation will improve the efficiency and speed of searches. The database will become an optional feature of ProMOL installation. With the database installed, ProMOL will first check for results in the database before performing a template-based alignment of the query against the library. The database will be manageable in size, since it will collate simple text files in the form of comma separated values.

Expanded motif options – Nearly 40% of all structures found in the PDB contains at least one metal ion. Currently motifs only include amino acids; future motifs will include metal ions and other prosthetic groups such as hemes, expanding the number of active sites that can be created and queried. It is anticipated that this will increase the predictive power of ProMOL.

JESS motifs – Motifs based on the JESS templates from the Catalytic Site Atlas are being created for ProMOL. These motifs include both alpha and beta carbon atom templates and full atom templates. Adding the JESS motifs to the ProMOL library will allow direct comparison of the ProMOL motifs with the Jess motifs.

*In vitro* testing – Several proteins without a function specified in the PDB entry for which strong possible motif matches were identified by ProMOL searches are currently being characterized biochemically [28]. *In vitro* testing will provide strong positive or negative support for the validity of results obtained from ProMOL searches.

## Conclusions

Due to the large number of protein structures determined by pipeline projects such as the Structural Genomics Initiative, there has been a large influx into the PDB of proteins with known structure but without clear functional annotation. ProMOL has been designed as a tool to aid in the determination of these structures’ functions. This is accomplished by comparing motif templates developed from the three-dimensional positions of active site residues in a protein of known function to the entire structure of a query protein. Improvements to ProMOL over the course of development have increased the program’s usability. The motif library currently contains over 180 motif templates based on CSA entries, and the resulting output has been improved by including RMSDs in addition to Levenshtein distances. In its current state, the true positive rate is about 60% with a false positive rate of 18%. The false positive results can be recognized by their significantly higher RMSD values compared to the true positives. Results varied greatly by EC class. In-depth studies of serine proteases revealed a true positive rate greater than 74% for enzymes in this class (Figure 3). An evaluation of the RMSD values for the serine proteases revealed a clear distinction in performance when all four EC numbers matched, as opposed to having three EC numbers matching (Figure 4). In addition, users can compare alignments visually within PyMOL to verify their findings. A performance comparison between ProMOL and 3DMSS-Sites, another template-based alignment program, revealed similar results with both programs. 3DMSS-Sites was more effective than ProMOL for the broad-brush-stroke level 1 searches, while ProMOL was more effective in the finer detailed level 3 searches (Figure 5). The similar performance of ProMOL and 3DMSS-Sites may well relate to the fact that both programs use site definitions from the CSA. The different results obtained with PDBSiteScan may reflect their use of motif definitions based on the SITE records in the PDB files.

## Availability and requirements

**Project Name:** ProMOL

**Project Homepage:**http://www.promol.org/

**Operating Systems:** Linux, Windows, Mac OS X

**Programming Language:** Python

**Other Requirements:** Python 2.6/2.7

**License:** GPL2

## Abbreviations

PDB: Protein data bank; CSA: Catalytic site atlas; EC: Enzyme commision; PSI: Protein structure initiative; RMSD: Root-mean-square deviation.

## Competing interests

The authors declare that they have no competing interests.

## Authors’ contributions

Corresponding author PAC initiated project work on ProMOL, along with BH and CW. MR implemented Levenshtein distances in ProMOL. AG and MO generated the results seen in Figures 3 and 4. MM implemented RMSD calculations and treewidgets in ProMOL. GD and PAC created the P motifs. PB performed test comparisons of ProMOL and 3DMSS-Sites. HK compared ProMOL with PDBSiteScan. TM compared ProMOL and BLAST results. CC contributed to software design and debugging. HJB contributed to the software design. All authors read and approved the final manuscript.

## Authors’ information

HJB: Dept. of Math and Computer Science, Dowling College, Shirley, NY, 11967, USA PAC, GD: School of Chemistry & Materials Science, Rochester Institute of Technology, Rochester, NY, 14624, USA. MO, AG, MM, MR, BH, CW, HK: Thomas H. Gosnell School of Life Sciences, Rochester Institute of Technology, Rochester, NY, 14624, USA. PB, TM: College of Health Sciences and Technology, Rochester Institute of Technology, Rochester, NY, 14624, USA. CC: Department of Computer Science, Rochester Institute of Technology, Rochester, NY, 14624, USA.
